# Supplementary material for: Designing lead-free antiferroelectrics for energy storage
Source: Nat Commun. 2017 May 30;8:15682. doi: 10.1038/ncomms15682 (PMC5499206; doi:10.1038/ncomms15682)
Supplement: Supplementary Information — Supplementary Figures, Supplementary Tables, Supplementary Notes and Supplementary References [file ncomms15682-s1.pdf]

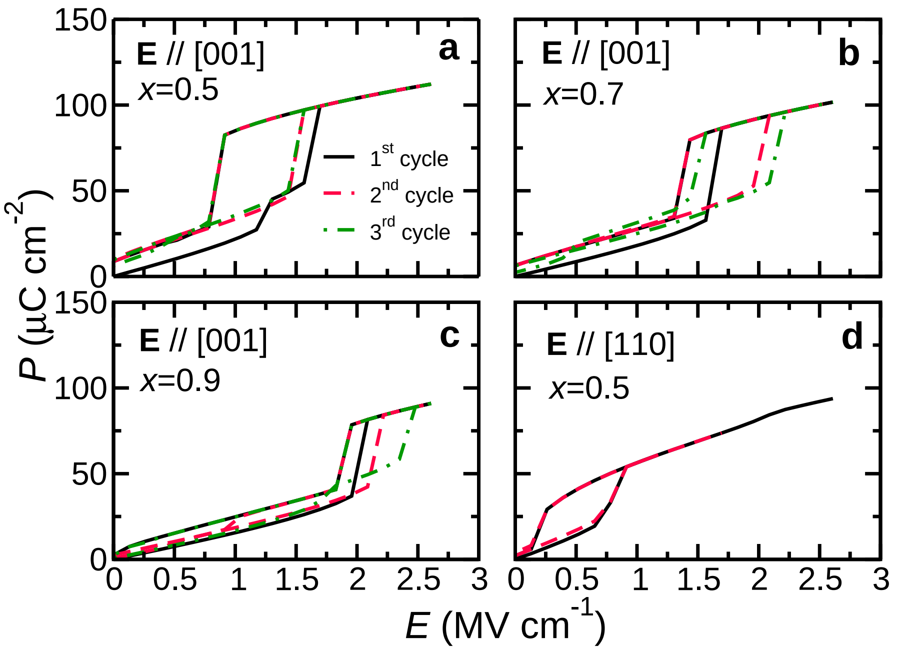

Supplementary Figure 1: **The P-E hysteresis loops of selected BNFO with additional cycles at 300 K.** (**a-c**) x=0.5, 0.7, and 0.9, respectively, with E along [001]. (**d**) x=0.5 with E along [110].

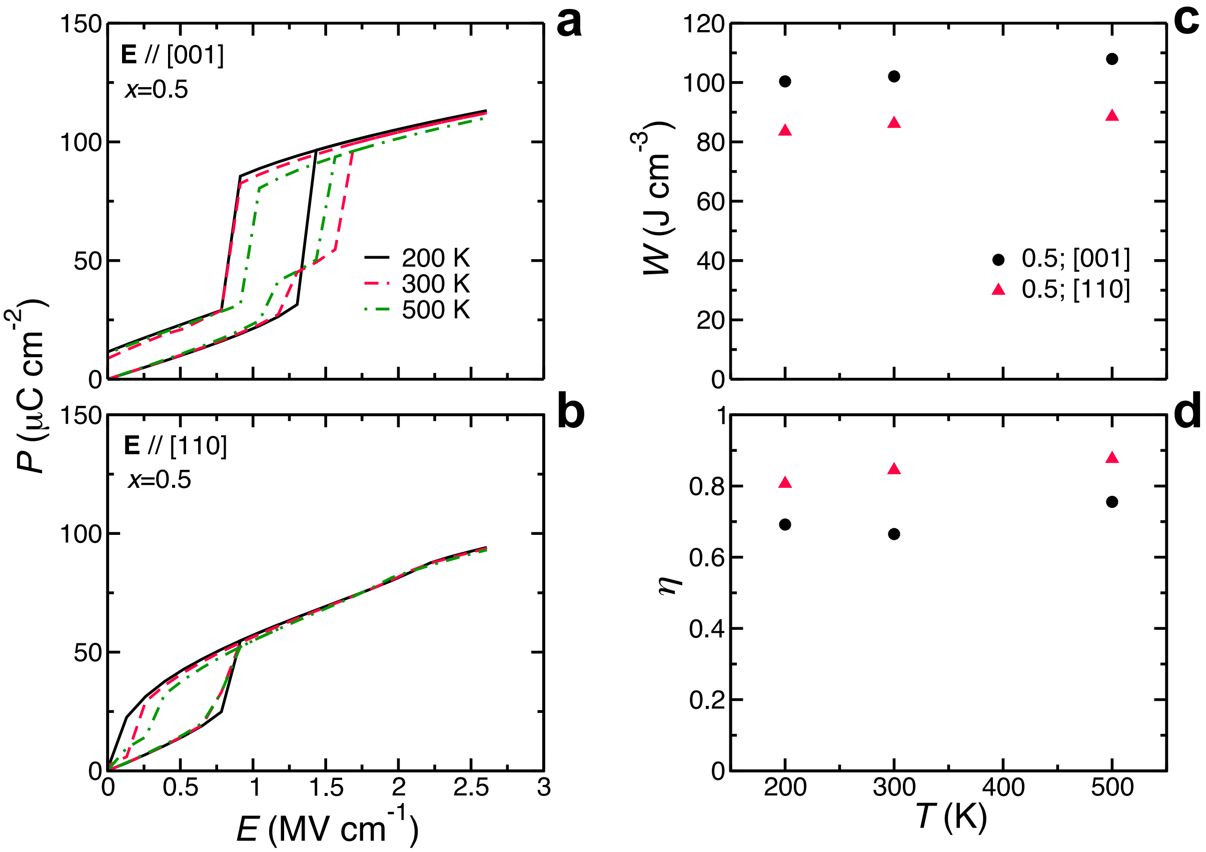

Supplementary Figure 2: **The effect of temperature on energy storage properties for BNFO with x=0.5.** (**a,b**) The P-E double hysteresis loops for electric field along the [001] and [110] direction, respectively, at temperatures of 200, 300, and 500 K. (**c,d**) The energy densities and efficiencies, respectively, at temperatures of 200, 300, and 500 K, for electric field along the [001] and [110] directions.

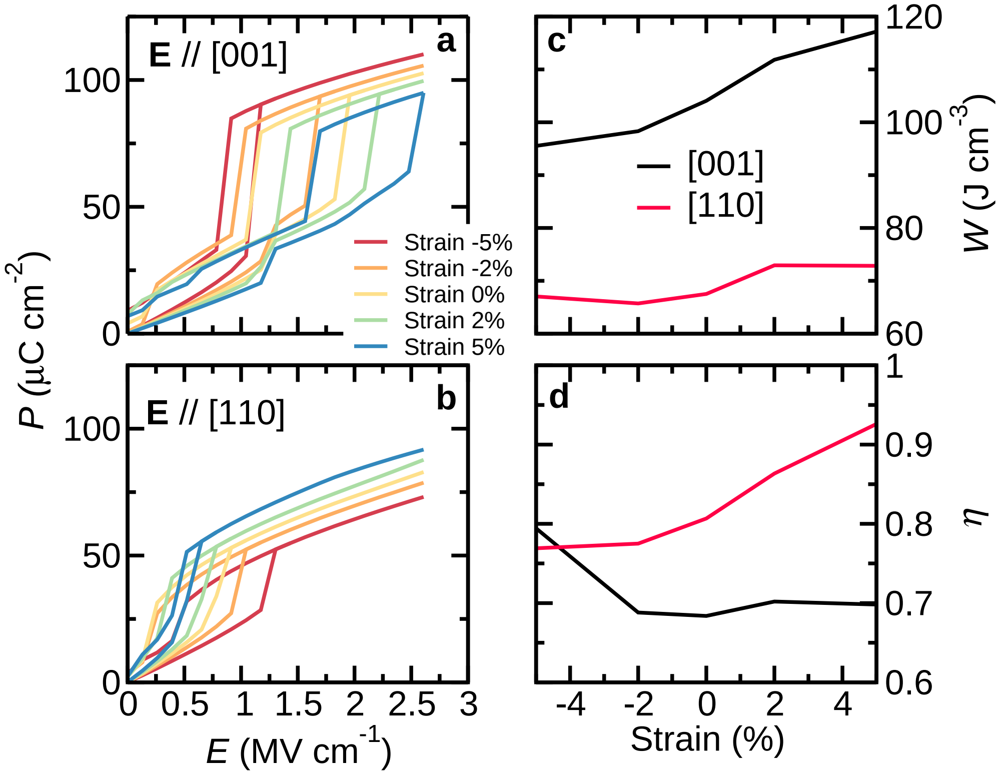

Supplementary Figure 3: **The effect of strain on storage properties.** (**a,b**) The P-E double hysteresis loops of BNFO with x=0.5 under electric field along the [001] and [110] direction, respectively, at 300 K, under an epitaxial strain of -5%, -2%, 0%, 2%, and 5%. (**c,d**) The corresponding energy density and efficiency as a function of strain.

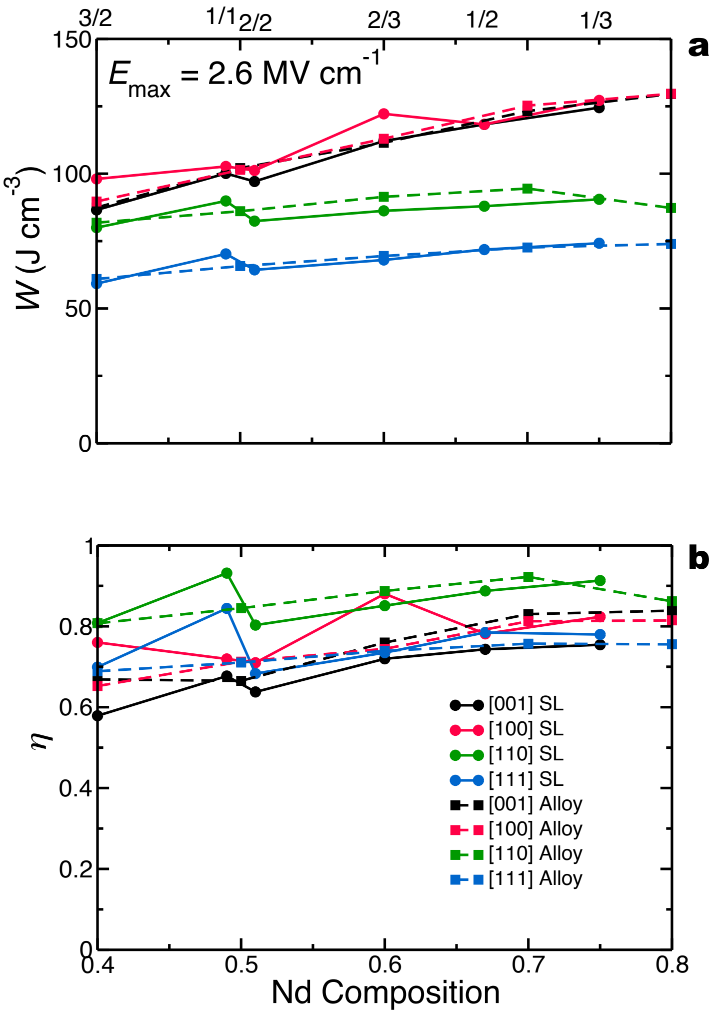

Supplementary Figure 4: **Comparison of the energy-storage properties between BNFO short-period superlattice and solid solution.** (**a,b**) Calculated energy densities and efficiencies at various Nd compositions (x=0.4–0.8) and under various electric field orientations, with the maximum applied E-field ($E_{\rm max}$) being 2.6 MV cm^{-1}.

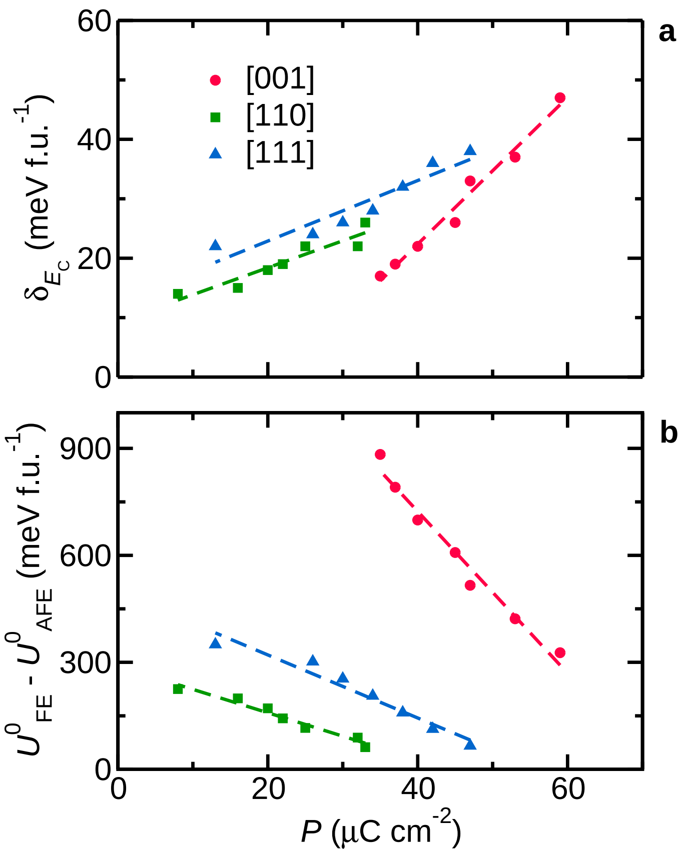

Supplementary Figure 5: **Correlation from various compositions.** (**a**) between the barrier δ_{E_C} and P^0_{FE} (polarization of the FE phase). (**b**) between $U^0_{\mathrm{FE}} - U^0_{\mathrm{AFE}}$ and P^0_{FE}. The dashed lines are linear fits.

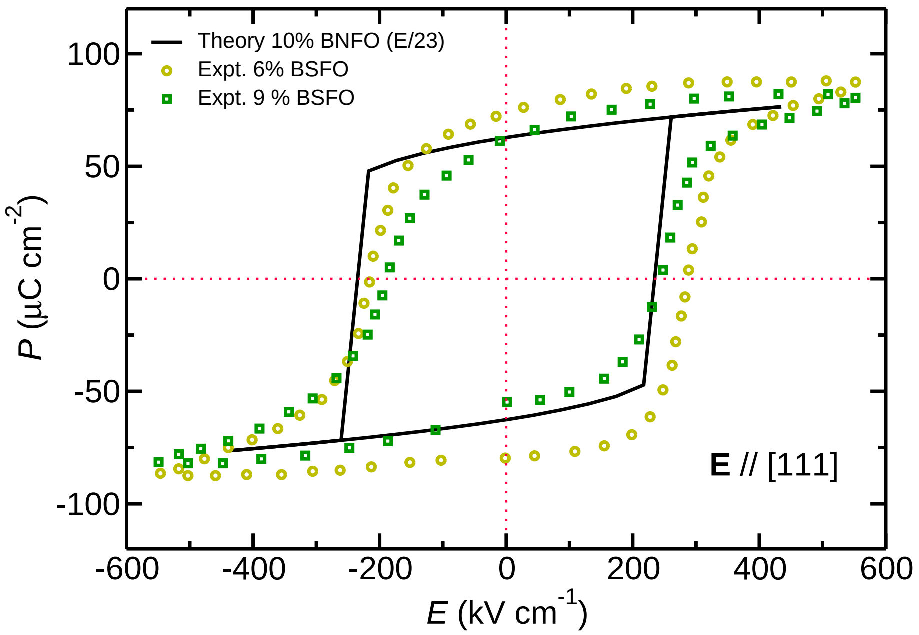

Supplementary Figure 6: **Calibration of the theoretical E-field.** P-E hysteresis loops of $Bi_{0.94}Sm_{0.06}FeO_3$ (BSFO), $Bi_{0.91}Sm_{0.09}FeO_3$ (BSFO) and $Bi_{0.9}Nd_{0.1}FeO_3$ (BNFO), under electric fields applied along the pseudocubic [111] direction at 300 K. The BSFO loops are experimental data[1], while the BNFO loop is our prediction once dividing the magnitude of the electric field by a factor of 23.

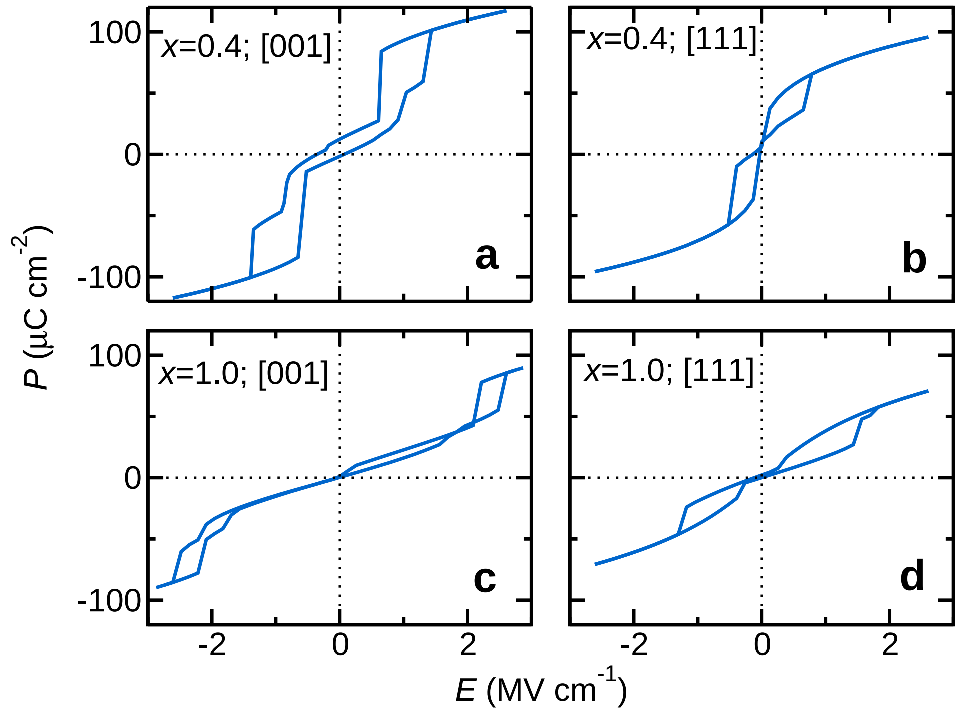

Supplementary Figure 7: **The bipolar P-E hysteresis loops of selected BNFO at 300 K.** (**a**) x=0.4 with electric field along [001]. (**b**) x=0.4 with electric field along [111]. **c**, x=1.0 with electric field along [001]. **d**, x=1.0 with E along [111].

Supplementary Table 1: **Structural information about the AFE, intermediate and FE states.**

These are the occurred structures during the simulated hysteresis loops for large E_{max}.

E direction	Description	AFE state	Intermediate state		FE state
	Space group	*Pnma*	Complex nanotwin		*P4mm*
[001]	Polarization	Anti-polar	Close to [001]		[001]
	AFD	$a^-a^-c^+$	Complex nanotwin		none
	Space group	*Pnma*	*Pc*		*P4mm*
[100]	Polarization	Anti-polar	$[uv0]$ $(u > v)$		[100]
	AFD	$a^-a^-c^+$	$a^-b^-c^0$		none
	Space group	*Pnma*	*Cc*	*Ima2*	*Amm2*
[110]	Polarization	Anti-polar	$[uuv]$ $(u > v)$	[110]	[110]
	AFD	$a^-a^-c^+$	$a^-a^-c^-$	$a^-a^-c^0$	none
	Space group	*Pnma*	Complex nanotwin		*R3c*
[111]	Polarization	Anti-polar	Close to [111]		[111]
	AFD	$a^-a^-c^+$	Complex nanotwin		$a^-a^-a^-$

Supplementary Table 2: **Parameters for the model.** The energies are in the units of meV per f.u. $\delta_{E_{\mathrm{down}}}$ is assumed to depend linearly on composition, and is obtained by least square fitting to the directly calculated energy densities and efficiencies for each E-field direction.

E direction	Nd composition	$U^0_{\mathrm{FE}} - U^0_{\mathrm{AFE}}$	δ^0	$\delta_{E_{\mathrm{C}}}$	$\delta_{E_{\mathrm{down}}}$	χ_{e}
	0.4	327	0.0	44.0	23.3	161
	0.5	422	0.0	35.9	22.2	158
	0.6	516	0.0	29.6	21.0	195
[001]	0.7	608	0.0	25.2	19.9	187
	0.8	699	0.0	21.7	18.7	205
	0.9	791	0.0	19.2	17.5	210
	1.0	883	0.0	18.0	16.4	209
	0.4	62	10.7	26.7	16.3	281
	0.5	89	9.4	23.3	15.8	278
	0.6	116	8.5	21.0	15.3	270
[110]	0.7	143	7.5	19.4	14.8	262
	0.8	171	6.3	17.7	14.3	249
	0.9	199	5.5	15.7	13.8	236
	1.0	225	5.5	15.0	13.3	233
	0.4	66	8.9	39.7	10.8	177
	0.5	113	2.0	34.7	11.9	182
	0.6	159	1.6	30.9	13.0	182
[111]	0.7	206	1.5	28.8	14.1	190
	0.8	254	1.2	26.1	15.2	192
	0.9	302	1.2	24.1	16.3	187
	1.0	350	1.9	22.0	17.3	185

Supplementary Note 1

Intermediate phases Let us now provide some information about the intermediate structures that appear from time to time in the hysteresis loops, in-between the AFE and FE states, and that generate some kinks in these loops. Note that we numerically found that these intermediate phases have relatively minor influence on the value of the energy density and efficiency. The structural description of the initial AFE phase, final FE state, and possible intermediate phases are summarized in Supplementary Table 1.

Interestingly, the intermediate phases were numerically found to mainly emerge during the charging process (increasing E).

For E along [001], one single intermediate phase connects the initial AFE phase and the final tetragonal *P4mm* phase with two successive first-order transitions. This phase is numerically found to be a complex nanotwin phase[2] for which the oxygen octahedral tiltings about the z-direction are modulated along the pseudo-cubic [001] direction.

For E along [100], there is also only one intermediate phase between the AFE and the *P4mm* FE phase, and this phase is also reached and then destabilized by first-order transitions. However, in contrast with the case of the field being applied along [001], this intermediate phase has the *Pc* space group, and is characterized by non-zero x and y components of the polarization and anti-phase tiltings.

Furthermore, two intermediate phases exist for E along [110], as shown in Supplementary Table 1. The AFE phase first transforms into the *Cc* phase via a first-order transition, followed by a second-order transition from *Cc* to the *Ima2* phase. The *Cc* phase has a polarization pointing along the $[uuv]$ direction (with $u > v$) and anti-phase tiltings about the $[u'u'v']$ direction ($u' > v'$). u is found to increase with E, while u', v and v' decrease. When v and v' become null, the structure smoothly transforms into the *Ima2* phase. The in-plane anti-phase tiltings then continue to decrease with field, and another second-order transition to an orthorhombic phase with space group *Amm2* takes place when all the tiltings vanish. Due to the second-order nature of the last two transitions, the polarization changes continuously.

Finally, when E is along the [111] pseudo-cubic direction, again there is one intermediate phase between the AFE phase and the final *R3c* FE state. This intermediate phase is another complex nanotwin phase that has been studied in ref. 3.

Supplementary Note 2

Repeatability of additional electric-field cycles In the manuscript, we have mentioned that, after the full first cycle is accomplished (charging and discharging), some final structures for vanishing field are not exactly the same as the initial AFE phase (at which the first cycle begins). One may thus wonder if the energy storage performance depends on the selected cycle. To answer such question, Supplementary Fig. 1 display the P-E curves of four representative cases for successive cycles.

The first three cases correspond to fields being along [001] but that differ between themselves by the selected Nd composition, while the fourth case is for a field applied along [110] for a single Nd composition of 0.5. In this fourth case shown in Supplementary Fig. 1d, one can see that the first and second cycles yield very similar hysteresis loop and thus lead to nearly identical energy storage properties. On the other hand, when E is along [001], the three studied successive full cycles can generate some differences, especially around the AFE-to-FE transition, either regarding the formation of the intermediate phase (Supplementary Fig. 1a) or shift of the AFE-to-FE E_{up} transition field (Supplementary Figs. 1b and 1c). The influence of these differences is, nevertheless, numerically found to be negligible on the energy density and efficiency, especially if the maximum field E_{max} is much higher than E_{up}.

Supplementary Note 3

Effect of temperature Let us now reveal how temperature affects the energy storage in (Bi,Nd)FeO_3 (BNFO). For that, Supplementary Figs. 2a and 2b show the P-E hysteresis loops of BNFO with x=0.5 with E being applied along the pseudo-cubic [001] and [110] directions, respectively, for three different temperatures, that are 200, 300 and 500 K. When E is along [001], one can see that no intermediate phase occurs at 200 K, unlike for 300 K and 500 K (where an additional kink is found for field of about 1.25 MV cm^{-1}), and increasing the temperature from 300 K to 500 K results in a shrinking of the energy loss. Such temperature-induced shrinking is also found for E applied along [110]. This slightly shrinking temperature-induced loss results in a slightly increasing W and η, as it can be seen in Supplementary Figs. 2c and 2d.

The effects of temperature revealed by Supplementary Fig. 2 can be understood by numerically determining and analyzing the T-dependency of some quantities in equations S11 and S12 (equations (5) and (6) of the manuscript): 1) $\delta_{E_{\text{down}}}$ is found to become closer to $\delta_{E_{\text{C}}}$ as the temperature increases, yielding an enhancement of both W and η; 2) polarization decreases with temperature, which is associated with a concomitant increase of $U^0_{\text{FE}} - U^0_{\text{AFE}}$ with temperature (because the AFE phase is more stable at higher temperature) and which is therefore also beneficial for energy storage purposes. Note that the relatively small effects of temperature on W and η in BNFO is also consistent with the small change in polarization for the temperature range (200–500 K) we investigate.

Supplementary Note 4

Strain effect We have reported in the manuscript the estimated energy storage performances for fully relaxed bulk systems. However, considering (001) epitaxial films, rather than relaxed bulks, can provide an additional handle for manipulating storage properties by playing with the strain the films are experiencing in the (x,y) plane. It is also appropriate for generating a large E_{max} electric field, since it is equal to the applied voltage divided by the (relatively small) thickness of the film. In Supplementary Figs. 3a and 3b, we show the variation of the 300 K P-E curves of BNFO with x=0.5 for different epitaxial strains varying from a -5% compressive strain to a +5% tensile strain, for fields applied along the pseudo-cubic [001] and [110] directions, respectively. In particular, for E along [001], increasing strain leads to 1) an increase of E_{up}; and 2) a decrease of the polarization of the final FE state at the largest investigated electric field. Items (1)-(2) are consistent with the

fact that the final structure under E along [001] is a *P4mm* tetragonal phase having an out-of-plane polarization, and that a tensile strain tends to disfavor such out-of-plane component and "pushes up the energy of the associated *P4mm* phase. Conversely, the opposite situation holds for items (1)-(2) when E is along [110] because the final *Ima2* FE phase possesses an in-plane polarization and therefore becomes more stable with increasing epitaxial strain.

Supplementary Figs. 3c and 3d show the energy density and efficiency as a function of strain. Distinctively different strain dependence is found between E along [001] and [110]. W for [001] field increases with strain (from compressive to tensile), while it is rather independent of strain for the [110] field. η, however, decreases at compressive strain then becomes insensitive to strain for [001] field, whereas a clearly increasing trend is found for the [110] field.

Here, the epitaxial strain plays a different role as compared to composition. Recall that higher composition reduces polarization, and the correlation in Supplementary Fig. 5 implies correspondingly larger $U^0_{\text{FE}} - U^0_{\text{AFE}}$ and smaller δ_{E_C}, which, therefore, enhances both W and η. For strain, the relationship between polarization and $U^0_{\text{FE}} - U^0_{\text{AFE}}$ is numerically found to remain valid (negatively correlated), but the correlation with δ_{E_C} becomes negative as well, so that, e.g., smaller polarization corresponds to larger $U^0_{\text{FE}} - U^0_{\text{AFE}}$ (the FE phase is disfavored) and larger δ_{E_C} (more energy loss). In other words, a competing effect from $U^0_{\text{FE}} - U^0_{\text{AFE}}$ and δ_{E_C} with respect to the change of strain sets in. Obviously, as it is shown in Supplementary Figs. 3c and 3d, $U^0_{\text{FE}} - U^0_{\text{AFE}}$ dominates the [001] case such that W increases with strain, whereas for the [110] field, the effects from $U^0_{\text{FE}} - U^0_{\text{AFE}}$ and δ_{E_C} are more of less balanced out for W, but the decreasing δ_{E_C} with strain causes the increasing

η. Nevertheless, we demonstrate that epitaxial strain can constitute an effective additional way to alter energy density and efficiency.

Supplementary Note 5

Superlattices versus solid solutions It is also interesting to check how superlattices (SL) behave, as compared with disordered solid solutions, for energy storage purposes. Here, we investigate BFO/NFO superlattices grown along the [001] direction and denote them as n/m when they possess n layers of BFO alternating with m layers of NFO along this growth direction. Note that some of the short-period SLs are hybrid improper ferroelectrics, that is they exhibit a small net polarization, P, along the in-plane [110] direction[4–6].

The comparisons between the disordered solid solutions and some SLs are illustrated in Supplementary Fig. 4 for the energy density and efficiency. In general, the SLs behave rather similarly to the random alloys, except that the compositional dependence for SLs is not as smooth as that for the solid solutions. The difference in energy density between these two types of materials is relatively less than their difference in efficiency, which arises from the similarity in the discharging P-E curves. Interestingly, SLs having the same overall composition but different number of n and m layers can also slightly differ in properties. For instance, for 1/1 and 2/2 (which corresponds to x=0.5), the 1/1 SL is better than the 2/2 SL regarding energy density and efficiency for all the studied orientations of the E-field. The 1/1 SL shows less energy-loss (smaller E_{up} and larger E_{down}) than that of 2/2 SL, likely due to the incomplete cancellation of its improper in-plane polarization.

Supplementary Note 6

Derivation of the model In order to derive a simple model, we make the following approximations: 1) the dielectric response, $\chi_{\rm e}$ is independent of the magnitude of the applied electric field, E, for a chosen direction of the electric field and is identical between the antiferroelectric (AFE) and ferroelectric (FE) states (note that this assumption is, in fact, confirmed by the effective Hamiltonian calculations reported in Fig. 3 of the manuscript); and 2) the AFE-to-FE and FE-to-AFE first-order transitions are abrupt, as illustrated in Fig. 1a of the manuscript, and that, for any given electric field, the AFE and FE phase are connected by an energetic path, along which the polarization continuously changes from its value in the AFE state (note that we still denote this state as AFE even when it acquires a small polarization as a result of applying an electric field) to its value in the FE phase. Different bridging structures i, each possessing their own polarization (denoted as P_i when there is no electric field), exist along this path. By using the same conventions as in the manuscript for physical quantities and calculating different areas shown in Fig. 1a of the manuscript, one can demonstrate that the energy density and efficiency are given by:

$$W = P^0_{\rm FE}E_{\rm down} + \frac{1}{2}\epsilon_0\chi_{\rm e}E^2_{\rm max} \tag{S1}$$

$$\eta = \frac{P^0_{\rm FE}E_{\rm down} + \frac{1}{2}\epsilon_0\chi_{\rm e}E^2_{\rm max}}{P^0_{\rm FE}E_{\rm up} + \frac{1}{2}\epsilon_0\chi_{\rm e}E^2_{\rm max}} \tag{S2}$$

where the maximum applied electric field $E_{\rm max}$ is larger than $E_{\rm up}$, and $P^0_{\rm FE}$ is the component of the polarization of the FE phase along the field direction as extrapolated to E=0.

Let us rewrite equations S1 and S2 in terms of physical quantities that are related to the aforementioned energetic path. For that, we express the energy of the AFE phase ($U_{\rm AFE}$), of the bridging

structures (U_i) and of the ferroelectric state (U_{FE}), all under an electric field as:

$$\begin{aligned} U_{\mathrm{AFE}} &= U_{\mathrm{AFE}}^0 - \frac{1}{2}\epsilon_0\chi_{\mathrm{e}}E^2 \\ U_i &= U_i^0 - P_iE - \frac{1}{2}\epsilon_0\chi_{\mathrm{e}}E^2 \\ U_{\mathrm{FE}} &= U_{\mathrm{FE}}^0 - P_{\mathrm{FE}}^0E - \frac{1}{2}\epsilon_0\chi_{\mathrm{e}}E^2 \end{aligned} \tag{S3}$$

where the last term on the right-hand side of each of the three equalities represents the energy associated with the field-induced electrical polarization. Fig. 1b of the manuscript schematizes the corresponding path for different magnitude of the electric field (for a chosen direction of this field). Let us now introduce the specific electric field, E_{C}, at which the FE and AFE phases have precisely the same energy. Equation S3 tells us that:

$$E_{\mathrm{C}} = (U_{\mathrm{FE}}^0 - U_{\mathrm{AFE}}^0)/P_{\mathrm{FE}}^0 \tag{S4}$$

Note that this E_{C} field is smaller than E_{up} (which is the field at which the AFE phase transforms into the FE phase) but larger than E_{down} (at which the FE phase reverts back to the AFE state). Next, we define the AFE-to-FE (respectively, FE-to-AFE) barrier, δ' (respectively, δ), as the energy difference between the saddle point and the AFE (respectively, FE) phase. δ' and δ are therefore equal at the E_{C} field.

Let us now consider the case of an electric field E that is smaller than E_{C}. In that situation, the FE-to-AFE barrier, $\delta_{E<E_{\mathrm{C}}}$, is the difference in energy between the bridging structure having the highest energy at this E field and the energy of the FE phase at this same E field (see Fig. 1b of the manuscript). Fig. 1b of the manuscript also tells us that the AFE-to-FE barrier at this field E is

the sum of $\delta_{E<E_{\rm C}}$ and the difference in energy between the FE and AFE phase at this E field. One thus has:

$$\text{For } E < E_{\rm C}: \text{AFE-to-FE barrier } \delta'_{E<E_{\rm C}} = U^0_{\rm FE} - U^0_{\rm AFE} - P^0_{\rm FE}E + \delta_{E<E_{\rm C}}$$
$$\text{FE-to-AFE barrier } \delta_{E<E_{\rm C}} \quad \text{(S5)}$$

We also assume that $\delta_{E<E_{\rm C}}$ varies in a linear fashion with the electric field, between its smallest value for E=0 (which is denoted as δ^0) and its largest value for $E_{\rm C}$ (denoted as $\delta_{E_{\rm C}}$, and which is the AFE-to-FE *and* FE-to-AFE barriers at $E_{\rm C}$), i.e.,

$$\delta_{E<E_{\rm C}} = \delta^0 + (\delta_{E_{\rm C}} - \delta^0)\frac{E}{E_{\rm C}} \quad \text{(S6)}$$

As a result, the $E_{\rm down}$ electric field can be simply expressed in terms of $E_{\rm C}$ as:

$$E_{\rm down} = \left(\frac{\delta_{E_{\rm down}} - \delta^0}{\delta_{E_{\rm C}} - \delta^0}\right) E_{\rm C} \quad \text{(S7)}$$

where $\delta_{E_{\rm down}}$ denotes the FE-to-AFE barrier at $E_{\rm down}$.

For the case of an electric field E that is larger than $E_{\rm C}$, the AFE-to-FE barrier, $\delta'_{E>E_{\rm C}}$, is now the energy difference between the bridging structure having the highest energy at this E field and the AFE phase at this E field (cf. Fig. 1b of the manuscript). The FE-to-AFE barrier at this field E is the sum of $\delta'_{E>E_{\rm C}}$ and the energy difference between the AFE and FE phase at this E field. As a result, we have:

$$\text{For } E > E_{\rm C}: \text{AFE-to-FE barrier } \delta'_{E>E_{\rm C}}$$
$$\text{FE-to-AFE barrier } \delta_{E>E_{\rm C}} = U^0_{\rm AFE} - U^0_{\rm FE} + P^0_{\rm FE}E + \delta'_{E>E_{\rm C}} \quad \text{(S8)}$$

$\delta'_{E>E_\mathrm{C}}$ is further assumed to vary linearly with the electric field, but with a slope that is exactly opposite to the one of equation S6, i.e.

$$\delta'_{E>E_\mathrm{C}} = \delta_{E_\mathrm{C}} - (\delta_{E_\mathrm{C}} - \delta^0)\frac{E - E_\mathrm{C}}{E_\mathrm{C}} \tag{S9}$$

when recalling that $\delta_{E_\mathrm{C}} = \delta'_{E_\mathrm{C}}$.

We also take δ'_{E_up} to be identical to δ_{E_down}, as both are limited by the thermal energy. With equation S9, the E_up electric field can therefore be related to E_C via:

$$E_\mathrm{up} = \left(\frac{2\delta_{E_\mathrm{C}} - \delta_{E_\mathrm{down}} - \delta^0}{\delta_{E_\mathrm{C}} - \delta^0}\right) E_\mathrm{C} \tag{S10}$$

Plugging equations S7 and S10 into equations S1 and S2, and then using equation S4 gives:

$$W = \left(\frac{\delta_{E_\mathrm{down}} - \delta^0}{\delta_{E_\mathrm{C}} - \delta^0}\right)(U^0_\mathrm{FE} - U^0_\mathrm{AFE}) + \frac{1}{2}\epsilon_0\chi_\mathrm{e}E^2_\mathrm{max} \tag{S11}$$

$$\eta = \frac{\left(\frac{\delta_{E_\mathrm{down}}-\delta^0}{\delta_{E_\mathrm{C}}-\delta^0}\right)(U^0_\mathrm{FE} - U^0_\mathrm{AFE}) + \frac{1}{2}\epsilon_0\chi_\mathrm{e}E^2_\mathrm{max}}{\left(\frac{2\delta_{E_\mathrm{C}}-\delta_{E_\mathrm{down}}-\delta^0}{\delta_{E_\mathrm{C}}-\delta^0}\right)(U^0_\mathrm{FE} - U^0_\mathrm{AFE}) + \frac{1}{2}\epsilon_0\chi_\mathrm{e}E^2_\mathrm{max}} \tag{S12}$$

These latter equations are precisely equations (6) and (7) of the manuscript.

Supplementary Note 7

Parameters for the model Let us now estimate the parameters entering the proposed model (note that these parameters depend on the chosen composition of the BNFO system as well as the directions of the applied electric fields, to be chosen here along the pseudo-cubic [001], [110], and [111] directions). Practically, the dielectric susceptibility is taken from the field variation of the

polarization of the corresponding FE phase (each chosen direction of the electric field generates a different FE state and thus a different $\chi_{\rm e}$). One can also see (Fig. 3 of the manuscript) that $\chi_{\rm e}$ of the AFE phase is similar to that of the FE phase, which is consistent with our assumption. To obtain the barriers for each field direction, we first extract a series of bridging structures from the AFE to the FE state by following the sweeps of the effective Hamiltonian Monte-Carlo simulations (mentioned in the Methods section of the manuscript) at a sufficiently large field ($\geq E_{\rm up}$) that allows direct AFE-FE transition. We then compute the energies of these bridging structures, as well as that of the AFE and FE states, for fields varying from E=0 to $E_{\rm up}$. We are then able to identify the specific field $E_{\rm C}$, at which $U_{\rm AFE} = U_{\rm FE}$, and to estimate the barrier $\delta_{E_{\rm C}}$. Similarly, $U^0_{\rm FE} - U^0_{\rm AFE}$ and the δ^0 barrier can be determined from the energetic path at E=0. The only unknown left in equations S11 and S12, that is $\delta_{E_{\rm down}}$, is assumed (for simplicity) to have a linear dependence on composition for each field direction, and is obtained by least square fitting to the directly calculated energy densities and efficiencies shown in Figs. 4a and 4b of the manuscript, respectively, for each direction. We numerically find that the compositional dependence of $\delta_{E_{\rm down}}$ is rather weak.

Supplementary Table 2 provides such parameters for different compositions of BNFO and for the selected direction of the electric fields. Note that a zero value of δ^0 in this Table is indicative that the energy of the corresponding FE state is higher than the energy of any bridging structure, under no applied field.

Interestingly, we also find trends between the value of some parameters and some physical quantities. For instance, Supplementary Fig. 5 shows overall linear relationships (by varying composi-

tion) between δ_{E_C}, $U^0_{FE} - U^0_{AFE}$ and the P^0_{FE} polarization of the FE state (note that P^0_{FE} is obtained as the difference of polarization between the FE and AFE states at E_{up}, as consistent with the schematization in Fig. 1a of the manuscript).

It is also interesting to realize that our predicted χ_e for pure $NdFeO_3$ is of the order of 200 at room temperature, which is fully consistent with extracted data related to the static dielectric response in ref. 7, which thus attests of the accuracy of the effective Hamiltonian simulations.

Supplementary Note 8

Calibration of the simulated electric field Equations S1 and S2 indicate that the energy density and efficiency are dependent on the magnitude of the electric fields. Moreover, it is well known that atomistic simulations have a tendency to overestimate critical fields, likely due to the so-called Landauer paradox[8]. Since we aim to provide quantitative predictions that can directly be compared with measurements, it is thus crucial to rescale here our simulated electric fields by a constant factor. To determine this factor, we computed the P-E curve of $Bi_{0.9}Nd_{0.1}FeO_3$ at 300 K and under electric fields applied along the [111] direction, and compare it with the available measured ones of $Bi_{0.94}Sm_{0.06}FeO_3$ and $Bi_{0.91}Sm_{0.09}FeO_3$ (BSFO) materials. It is expected that these three materials should exhibit very similar hysteresis loops because properties of rare-earth-doped BFO systems were experimentally found to mostly only depend on the average ionic radius[9] (the average ionic radius of BNFO with x=0.1 is in-between those of BSFO with x=0.06 and x=0.09).

Supplementary Fig. 6 reveals that rescaling down the mimicked electric fields by a factor of 23 provides a hysteresis loop that agrees rather well with experimental data. Such rescaling is systematically used in our predictions in the manuscript and SI.

Supplementary Note 9

Bipolar hysteresis loop For a few representative cases, i.e. x=0.4 or 1.0 with E along [001] or [111], we simulate the bipolar hysteresis loops of BNFO, as shown in Supplementary Fig. 7. Positive and negative E yield rather similar shape of the P-E curves (of opposite polarization), which thus yield similar energy density and efficiency.

Supplementary Note 10

Morphotropic phase boundary In this section, we briefly discuss the energy storage properties near the morphotropic phase boundary (MPB) between the FE and AFE phases. We have predicted in ref. 3 that the MPB in BNFO consists of complex nanotwins phases. Structurally, these complex phases are bridging phases between the rhombohedral $R3c$ ground state for small Nd composition and the orthorhombic $Pnma$ ground state for large Nd concentration. They were found to possess small polarizations, which is detrimental for reaching high-energy density and efficiency.

Although a single-phase MPB does not show promising storage properties according to our calculations (not shown here), it is interesting to realize that high-energy density and efficiency have been realized in the MPB of $(Bi_{1/2}Na_{1/2})_{0.9118}La_{0.02}Ba_{0.0582}(Ti_{0.97}Zr_{0.03})O_3$ (BNLBTZ) relaxor

films[10]. We believe that this is likely due to the coexistence of multiple energetically competing phases that may enhance the dielectric response, as reported in ref. 9 and as consistent with equation S1.

Supplementary References

1. Kan, D., Anbusathaiah, V. & Takeuchi, I. Chemical substitution-induced ferroelectric polarization rotation in $BiFeO_3$. *Adv. Mater.* **23**, 1765–1769 (2011).

2. Prosandeev, S., Wang, D., Ren, W., Íñiguez, J. & Bellaiche, L. Novel Nanoscale Twinned Phases in Perovskite Oxides. *Adv. Funct. Mater.* **23**, 234–240 (2013).

3. Xu, B., Wang, D., Íñiguez, J. & Bellaiche, L. Finite-temperature Properties of Rare-Earth-Substituted $BiFeO_3$ Multiferroic Solid Solutions. *Adv. Funct. Mater.* **25**, 552–558 (2015).

4. Rondinelli, J. M. & Fennie, C. J. Octahedral rotation-induced ferroelectricity in cation ordered perovskites. *Adv. Mater.* **24**, 1961–1968 (2012).

5. Mulder, A. T., Benedek, N. A., Rondinelli, J. M. & Fennie, C. J. Turning ABO_3 Antiferroelectrics into Ferroelectrics: Design Rules for Practical Rotation-Driven Ferroelectricity in Double Perovskites and $A_3B_2O_7$ Ruddlesden-Popper Compounds. *Adv. Funct. Mater.* **23**, 4810–4820 (2013).

6. Xu, B. *et al.* Hybrid Improper Ferroelectricity in Multiferroic Superlattices: Finite-Temperature Properties and Electric-Field-Driven Switching of Polarization and Magnetization. *Adv. Funct. Mater.* **25**, 3626 (2015).

7. Chanda, S., Saha, S., Dutta, A. & Sinha, T. P. Raman spectroscopy and dielectric properties of nanoceramic $NdFeO_3$. *Mater. Res. Bull.* **48**, 1688–1693 (2013).

8. Daumont, C. *et al.* Strain dependence of polarization and piezoelectric response in epitaxial $BiFeO_3$ thin films. *J. Phys. Condens. Matter* **24**, 162202 (2012).

9. Kan, D. *et al.* Universal Behavior and Electric-Field-Induced Structural Transition in Rare-Earth-Substituted $BiFeO_3$. *Adv. Funct. Mater.* **20**, 1108–1115 (2010).

10. Peng, B. *et al.* Giant Electric Energy Density in Epitaxial Lead-Free Thin Films with Coexistence of Ferroelectrics and Antiferroelectrics. *Adv. Electron. Mater.* **1**, 1500052 (2015).
